# Application and evaluation of the global trigger tool approach to adverse drug event monitoring in the high-risk elderly inpatients with multiple chronic diseases

**DOI:** 10.3389/fphar.2025.1594176

**Published:** 2025-06-20

**Authors:** Na Li, Xiao-Min Lv, Chen-Yang Jiao, Dong-Li Zhang, Lin-Wei Chen, Yi-Ling Chang, Jin-Fang Song

**Affiliations:** ^1^ Department of Pharmacy, Taizhou People’s Hospital, Jiangsu, Taizhou, China; ^2^ Department of Clinical Pharmacy, Affiliated Hospital of Jiangnan University, Jiangsu, Wuxi, China; ^3^ Jiangsu Key Laboratory of New Drug Research and Clinical Pharmacy, Xuzhou Medical University, Jiangsu, Xuzhou, China

**Keywords:** global trigger tool, elderly, multiple chronic diseases, adverse drug events, inpatients

## Abstract

**Objective:**

To establish a Global Trigger Tool (GTT) method suitable for monitoring adverse drug events (ADEs) in the high-risk elderly inpatients with multiple chronic diseases, and to evaluate its sensitivity, specificity and feasibility.

**Methods:**

A total of 38 triggers were established by searching the literature and combining the characteristics of elderly hospitalized patients with multiple chronic diseases in Taizhou People’s Hospital. A total of 480 elderly patients with multiple chronic diseases were sampled from January to December 2023, and the cases were reviewed. Adverse event grades were determined, and drug classes and organ-systems involved were analyzed; binary logistic regression and Receiver Operating Characteristic curves were adopted for analysis.

**Results:**

Among the 480 cases, 123 cases were detected as having one or more positive triggers. ADEs occurred in 65 patients, with a total of 93 occurrences of ADEs; the highest number of ADE cases was observed in the administration of cardiovascular drugs, with 36 cases (38.71%). The highest organ-system involved in ADE was metabolic and nutritional disorders, with 47 cases (50.54%). The number of ADEs occurring in 1,000 patient-days was 22.90. The number of ADEs occurring in 100 patients was 19.38. Using binary logistic regression analysis, the risk factors were age and number of positive trigger detections for predicting the occurrence of ADEs. The GTT method had a sensitivity of 78.46%; specificity of 82.65%; compliance rate of 82.00%; Kappa value of 44.40%; and the Positive Predictive Value (PPV) was 41.46%.

**Conclusion:**

The GTT method has high sensitivity and specificity and is feasible; it has a relatively high PPV and is suitable for detecting ADEs in the high-risk elderly inpatients with multiple chronic diseases.

## 1 Introduction

With the continuous development of society, China has entered the stage of accelerated population aging, and the demand for social services in this stage is increasing, especially medical services. Chronic diseases now account for 88.5% of mortality in China, with 55%–98% of elderly people affected. Notably, 50%–80% of elderly patients present with multimorbidity requiring complex medication regimens (Abdu et al., 2025; Dong and Zheng, 2022; Gao et al., 2024). The proportion of polypharmacy among elderly chronic disease patients in the community in China ranges from 33.1% to 75.3% (World Health Organization, 2019), and the proportion of polypharmacy among inpatients ranges from 48.0% to 95.7% (Zeng et al., 2019). Polypharmacy (≥5 medications) can increase the risk of Adverse Drug Event (ADE) by 88% (Maher et al., 2014). This medication complexity may lead to an increase in drug-related problems (DRPs), poor patient adherence to medication, increased readmission rates, and prolonged hospital stays (Ulley et al., 2019). Hu et al. emphasize the need to prioritize actions to benefit this particularly vulnerable population (Hu et al., 2020).

In 2003, a new method for monitoring ADEs was introduced in the United States-the Global Trigger Tool (GTT) method. It is a method that looks for triggers during the review of cases and purposefully locates ADE-related content in the case so that clinicians can further analyze and identify ADEs, and it can effectively compensate for the shortcomings of other methods (Yu et al., 2023). Several foreign studies have proved that the GTT method can monitor the incidence of ADEs more effectively than the incident reporting system (Hibbert et al., 2023; Moraes et al., 2023). And GTT method has higher sensitivity and accuracy than other methods in identifying all ADEs in adult hospitalized patients and ADEs that are more harmful to patients. Currently, GTT method has replaced other monitoring methods in foreign countries and becomes a daily monitoring tool in hospitals. In contrast, research on the GTT method has only begun in China since 2012. Up to now, the domestic research on GTT method is still in the stage of exploration and improvement, and has not been widely used. And the research object is mostly focused on adult and pediatric patients, with fewer elderly patients. So far, there is no research on elderly hospitalized patients with multiple chronic diseases (Brösterhaus et al., 2022; Meng et al., 2024), especially for high-risk elderly groups (i.e., taking >5 drugs or engaging in substance abuse). Compared with foreign countries, the domestic GTT method system is still immature and the positive trigger detection rate is not high, while the simplification in the number of triggers in foreign countries will make PPV increase (Dotta et al., 2024).

Given the strong practicality and feasibility of the GTT method for monitoring ADE, this study proposes to use the GTT method to monitor ADEs in high-risk elderly hospitalized patients with multiple chronic diseases, to determine the drug class, severity of ADEs and organ-systems involved in this group of patients, and to analyze and evaluate the results. Meanwhile, the reality and feasibility of the GTT method were further confirmed, with a view to providing an effective basis and security for the medication safety of high-risk elderly hospitalized patients with multiple chronic diseases.

## 2 Materials and methods

### 2.1 Objectives

Elderly patients who were hospitalized in Taizhou People’s Hospital from January to December 2023 were selected for this study.

Inclusion criteria

• hospitalization duration ≥2 days
• age ≥65 years
• suffering from two or more chronic diseases.
• more than five medications.

Exclusion criteria

• patients with oncologic diseases
• incomplete clinical data.

### 2.2 Sample size calculation

**FIGURE 1:**
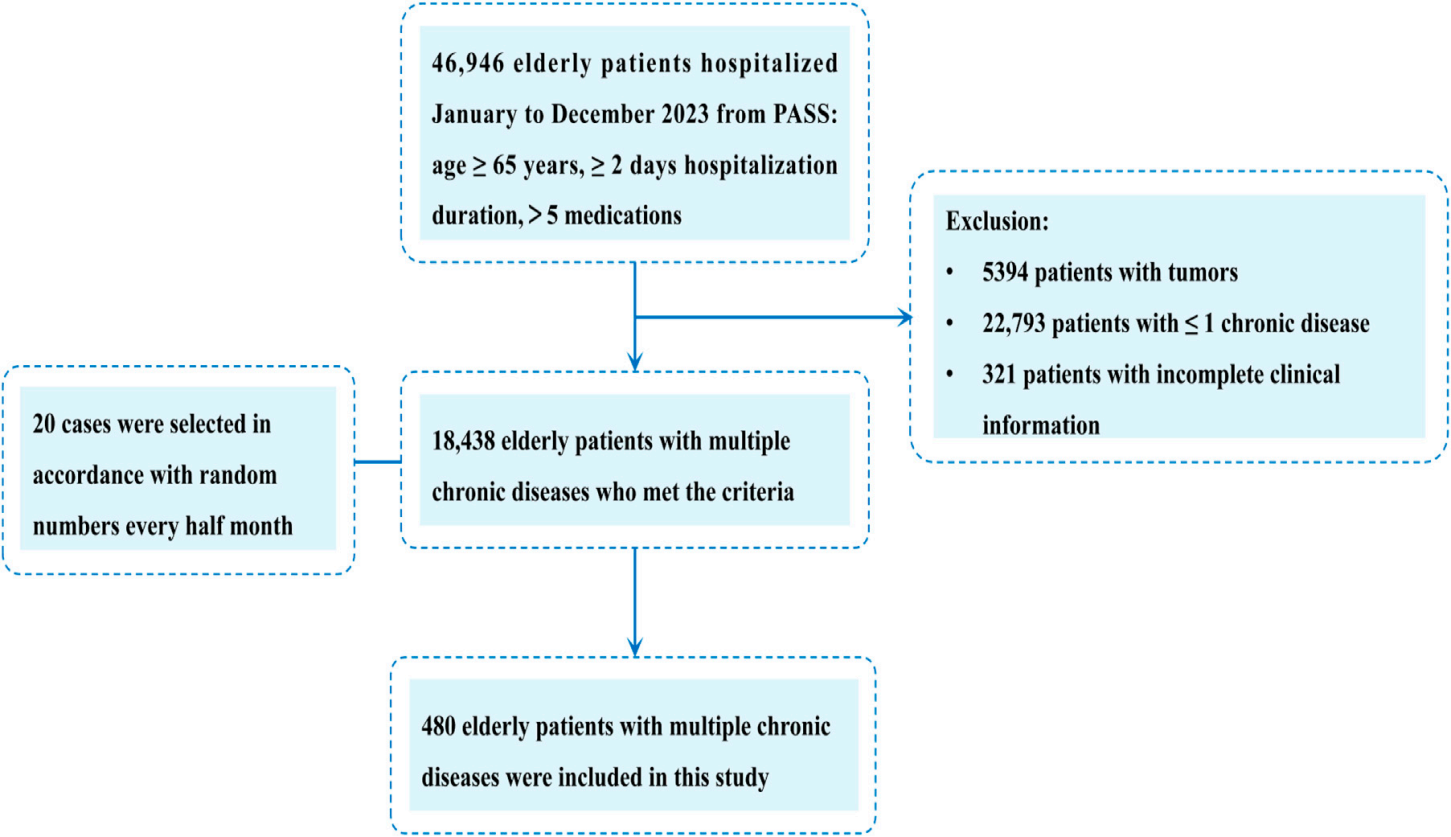
Flowchart illustrating the process of study identifcation, inclusion, and exclusion. PASS, Prescription Automatic Screening System.

Cases were selected according to the inclusion and exclusion criteria. The sample size was examined with G*Power 3.1.9.7 software (Faul et al., 2009). Combining previous literature and preliminary experimental results, the total detection rate of GTT was about 20%, and the effect size d was taken as 0.2 according to Cohen’s calculation. The two-sided significance level was taken as 0.05, and the statistical efficacy was taken as 95%. The results showed that inclusion of 327 patients could have sufficient statistical efficacy. Based on the above analysis, the total sample size planned for inclusion in this study was 480 patients. After determining the sample size, Systematic Time-based Random Sampling was used. A random number between 0 and 1 was generated using the Excel RAND () function every half-month, and the first 20 cases were selected as samples according to the order of the random numbers, with 40 cases selected every month, and a total of 480 cases were selected (Figure 1).

### 2.3 Trigger selection and formulation

According to the data, the trigger is an important embodiment in determining the effectiveness of the GTT method for monitoring ADEs. When applied in different regions or groups, the trigger should be developed according to the actual situation of ADEs and the clinical use of medication in this hospital, so that corresponding measures can be taken for different types of adverse events, which can have an effective improvement in the detection rate of ADEs (Liu et al., 2020; Pandya et al., 2020).

This study was based on the triggers of the medication module in the second edition of the GTT white paper and the triggers used in other related literature studies (Hu et al., 2020; Hu et al., 2019), which were adjusted, supplemented, and deleted based on the indicator ranges of biochemical tests and the medication catalog of the hospital, and then based on the common ADRs of elderly patients (Huang, 2017), the risk points of the potential inappropriate use of medication catalogs (Yan et al., 2015), and the prescribing habits of the hospital. A total of 38 triggers were finally developed for ADE detection in the hospital (Table 2). Criteria for positive trigger detection: the presence of ≥1 trigger in the patient.

### 2.4 ADE judgment

Currently, the method used by China’s Adverse Drug Reaction Monitoring Center to determine ADE is the five-level standard, which has significant differences in specificity and sensitivity (Wei and Xie, 2012). In comparison, the Naranjo rating method is more conducive to the detection of ADEs, especially rare ADEs, so in this study, the Naranjo rating method was used to determine ADEs (Naranjo et al., 1981).

The Naranjo scale consists of 10 items, and the degree of certainty of causality is categorized into 4 levels: “definite”, “probable”, “possible” and “doubtful”. And determined by the score: “definite” ≥9 points; “probable” for 5-8 points; “possible” for 1- 4 points; “doubtful” ≤0 points (as shown in Supplementary Table S1). According to the Naranjo scale standardization process, cases with a score of ≤0 (‘doubtful’) were considered as non-drug related events and were not included in the ADE analysis.

### 2.5 ADE grading

According to the second edition of the GTT White Paper, it is recommended that the medication error grading criteria developed by the National Coordinating Council For Medication Error Reporting And Prevention (NCC MERP) be used to grade the severity of ADEs. Since grades A-D did not cause substantial harm to patients and had little impact, and the white paper was mainly based on grades E-I, the study of ADEs grading in this study is based on grade E-I adopted by the white paper (Table 1).

**TABLE 1:** NCC MERP medication error grading criteria (Grades E-I).

Error grading	Judgments
Grade E	Temporary harm to the patient requiring intervention
Grade F	Temporary harm to the patient requiring hospitalization or prolonged hospitalization
Grade G	Permanent harm to the patient
Grade H	Intervention required to maintain life
Grade I	Patient death

NCC MERP, the National Coordinating Council For Medication Error Reporting And Prevention.

### 2.6 Case review

According to the guidelines outlined in the IHI white paper, the review panel including four members was established. All members of the review panel underwent prior standardised review training. Two junior reviewers with clinical backgrounds (one clinician and one clinical pharmacist) conducted independent reviews of the cases, including basic information, medical history records, medical orders, and laboratory test results. Two senior-level with clinically relevant backgrounds (one clinician and one clinical pharmacist) were responsible for addressing questions during the review process, verifying ADEs, and conducting grading and preventability assessments. The average time for the review process was limited to 30 min per record.

Test results, medical orders, medical records, and surgical records from the 480 cases screened were reviewed by primary reviewers. The trigger detection was determined to be a positive criterion, and basic patient information was also collected. Problems encountered during the review process needed to be reported to the senior reviewer. Subsequently, discussion and analysis were carried out, and it was sufficient for three members to agree on the judgment results.

Patients were assessed for the occurrence of ADEs, and the severity of ADE was graded by recording the relevant drug class and organ-system involved. Trigger positive detection rate, the number of ADEs occurring in 1,000 patient-days, the number of ADEs occurring in 100 patients, and the incidence of ADEs in patients were calculated. The trigger positive detection rate is the number of positive triggers/the total number of medical records × 100%, the larger the trigger positive detection rate, the higher the sensitivity of the trigger. The PPV of ADE is the ratio of the number of ADE cases to the frequency of positive triggers, and the closer the PPV is to 1, the better the specificity of the trigger is, and the more reasonable it is.

### 2.7 Statistical method

Excel 2016 and SPSS 27.0 software were used for statistical analysis. Trigger-positive cases (≥1 trigger trigger defined as positive) were identified by the GTT tool and subsequently analysed by logistic regression for association between trigger-positive status and ADE occurrence. Measurement data, which conformed to normal distribution were expressed as (‾x ± s) and compared using the two independent samples t-test; those which did not conform to normal distribution were expressed as M (Q1 - Q3) and compared using the Mann-Whitney U-test; and counting data were expressed as n (%) and compared using the chi-square test. Risk factors for the occurrence of ADE were analyzed by binary logistic regression, and the predictive value of the model was assessed by the Area Under the Curve (AUC) using statistically different indicators as the independent variables. The AUC values ranged from 0.5 to 1.0, with an AUC of 0.5–0.75 being acceptable, and an AUC >0.75 indicating that the model showed good discrimination. *P* < 0.05 was considered statistically significant.

## 3 Results

### 3.1 Baseline characteristics

In this study, a total of 480 cases from Taizhou People’s Hospital from January to December 2023 that matched the criteria were selected. Among them, 263 cases were male and 217 cases were female. The number of chronic disease types ranged from 2 to 10. The most frequent comorbidities were: Hypertension (34.45%, n = 392), Type 2 Diabetes (27.68%, n = 315), Coronary Artery Disease (16.87%, n = 192), Hyperlipidaemia (8.17%, n = 93),Chronic Kidney Disease (4.13%, n = 47). Age was in the range of 65–96 years with a mean age of 74.29 years. The number of days of hospitalization was in the range of 2–24 days, and the mean number of days of hospitalization was 8.38 days. The number of medications used ranged from 5 to 46, with an average of 16.05 medications.

### 3.2 Triggers

Among the 38 triggers, 27 triggers were detected as positive, of which 18 had PPV values higher than 50.00%, totaling 204 positive triggers. “Blood potassium <3.5 mmol/L” had the highest number of positive detections, with a total of 43 cases, accounting for 21.08% (43/204); followed by “platelet count <125 × 10^9^/L”, with 21 cases; and ranking third was “edema” with 20 cases. Eleven triggers were negative, including “fecal *C. difficile* positive”, “flumazenil use”, “naloxone use”, “excessive sedation/hypotension/falls”, “abrupt discontinuation”, “use of protamine sulfate”, “use of epinephrine or glucocorticoids”, “dehydration”, “urinary retention”, “vancomycin blood drug concentration >10 mg/L”, “gentamicin blood drug concentration >10 mg/L” (Table 2).

**TABLE 2:** Positive trigger detections.

No.	Trigger	Interpretation	Positive triggers	ADE cases	PPV (%)
1	Fecal test positive for Clostridium difficile	Antibiotic-associated diarrhea	0	0	0.00
2	APTT>39 s	Heparin overdose	10	7	70.00
3	INR>1.2 s	Warfarin overdose	2	2	100.00
4	BG < 3.9 mmol/L	Use of insulin or oral hypoglycemic drugs	10	7	70.00
5	CK > 164 U/L; BUN>9.5 mmol/L; SCr(male) > 111 μmol/L; SCr(female) > 81 μmol/L	Use of renal impairment drugs	9	5	55.56
6	Use of vitamin K	Excessive bruising, Gastrointestinal bleeding, hemorrhagic stroke or large hematoma	1	1	100.00
7	Use of flumazenil	Rescue sedation causing severe hypotension or significant prolonged sedation	0	0	0.00
8	Use of naloxone	Rescue from opioid poisoning	0	0	0.00
9	Hypersedation/hypotension/falls	Use of sedative-hypnotics/hypotensive drugs	0	0	0.00
10	Drug withdrawal	ADE requiring discontinuation of medication for control or ADE due to abrupt discontinuation of medication	0	0	0.00
11	ALT>35 U/L; AST>40 U/L; ALP>125 U/L; TBIL>23 μmol/L	Use of drugs for liver damage	1	1	100.00
12	K > 5.3 mmol/L	Hyperkalemic drugs used	5	3	60.00
13	K < 3.5 mmol/L	Hypokalemic drugs used	43	24	55.81
14	Na>147 mmol/L	Hypernatremic drugs used	7	4	57.14
15	Na<137 mmol/L	Hyponatremic drugs used	17	4	23.53
16	WBC<3.5 × 10^9^/L	Use of leukopenic drugs	5	3	60.00
17	PLT<125 × 10^9^/L	Use of Thrombocytopenic Drugs	21	7	33.33
18	TC > 5.2 mmol/L	Drug-induced cholesterol elevation	11	6	54.54
19	HGB>175 g/L	Use of recombinant human erythropoietin	5	0	0.00
20	TSH<0.56 mU/L,TH > 152.52 nmol/L	Use of hyperthyroid-causing drugs	1	0	0.00
21	TSH>5.91 mU/L,TH < 69.97 nmol/L	Use of hypothyroidizing drugs	1	0	0.00
22	Use of montelukast/bifidobacterium trifidum/bacillus subtilis/oral metronidazole	Rescue of antibiotics and other drug-associated diarrhea	8	1	12.50
23	Use of protamine sulfate	Rescue Heparin Overdose	0	0	0.00
24	Use of epinephrine or glucocorticoids	Rescue of drug-induced anaphylaxis	0	0	0.00
25	Use of glycerol enema/lactulose oral solution	Rescue from medicinal constipation	8	0	0.00
26	Use of metoclopramide	Rescue vomiting symptoms from surgery, chemotherapy or drug use	3	2	66.67
27	Transfer to intensive care unit/re-admission within 30 days	Patient deterioration	7	0	0.00
28	Rash/Itch	Use of drugs causing skin reactions	2	2	100.00
29	Agitation/restlessness/sleep disturbance/depression/delirium/suicidal ideation/suicidal behavior	Use of drugs causing the occurrence of symptoms in the psychiatric system	1	1	100.00
30	Drowsiness/Vertigo/Headache/Dizziness/Memory Impairment/Attention Deficit Disorder/Tremor/Myalgias	Use of drugs causing nervous system reactions	2	2	100.00
31	Cognitive impairment	Use of drugs causing confusion or cognitive impairment	1	0	0.00
32	edema	Use of drugs causing oedema	20	8	40.00
33	Dehydration	Use of diuretics	0	0	0.00
34	Urinary retention	Use of urinary retention medications	0	0	0.00
35	Digoxin Blood Concentration >2 ng/mL	Digoxin overdose	2	2	100.00
36	Vancomycin Blood Concentration >10 mg/L	Vancomycin overdose	0	0	0.00
37	Gentamicin blood concentration >10 mg/L	Gentamicin overdose	0	0	0.00
38	Joint pain	Drug-induced joint disorders	1	1	100.00
Total			204	93	45.59

BG, blood glucose; APTT, activated partial thromboplastin time; INR, international normalized ratio; CK, creatine kinase; BUN, blood urea nitrogen; SCr, serum creatinine; ALT, alanine aminotransferase; AST, aspartate transaminase; ALP, alkaline phosphatase; TBIL, total bilirubin; WBC, white blood cell count; PLT, platelet; TC, total cholesterol; HGB, hemoglobin; TSH, thyroid stimulating hormone; TH, thyroid hormone.

*PPV, ADEs/positive triggers.

### 3.3 Adjudication of ADE and application of the GTT method

The relationship between the number of trigger positives and the occurrence of ADEs was determined according to the Naranjo method. The results indicated that there were 93 ADEs among 65 cases, of which 2 cases were “definite”, 5 cases were “probable”, and 86 cases were “possible”. Two ‘doubtful’ cases were excluded based on the standardised procedure of the Naranjo scale. There were 46 cases of 1 ADE, accounting for 70.77% (46/65). All 95 ADE events and their corresponding Naranjo causality grades are listed in Supplementary Table S2.

Among 480 patients, 123 triggered positive trigger detection. 65 cases (13.54%) were confirmed to have at least one ADEs. Of these, 51 cases tested positive for trigger detection and experienced adverse events; 72 cases were detected positive for the trigger but ADEs did not occur; 14 cases were not detected positive for the trigger but ADEs occurred; and 343 cases did not test positive for the trigger and ADEs did not occur (Table 3). The application of the GTT method was judged on the basis of the above data, and it was calculated that: the sensitivity was 78.46% (51/(51 + 14)); the specificity was 82.65% (343/(343 + 72)); the compliance rate was 82.00% ((51 + 343)/480); the Kappa value was 44.40%; and the PPV was 41.46% (51/123). The results revealed high sensitivity and specificity with good compliance.

**TABLE 3:** Relationship between triggers detection and Naranjo evaluation.

Triggers detection	Naranjo method for determining ADEs		Total
	Number of patients with at least one ADE	Number of patients without a single ADE	
Positive	51	72	123
Negative	14	343	357
Total	65	415	480

ADE, Adverse drug event.

The higher the sensitivity and specificity, the better the authenticity of the GTT method; the lower it is, the method has no clinical application prospect. The higher the compliance rate and Kappa value, the higher the feasibility of the GTT method; the lower it is, the method has a certain amount of error. Thus the GTT method has a high degree of authenticity and a high degree of feasibility. The PPV is not so high, probably due to the uncertainty of the PPV in relation to whether or not an ADE occurs with a positive detection. Adjustment is still needed on the trigger as a way to increase the PPV.

### 3.4 Classification of ADEs

ADEs occurring in the cases were classified according to the NCC MERP medication error grading criteria (grades E-I). The results showed that among 93 cases, there were 90 cases of grade E, accounting for 96.77% (90/93), and 3 cases of grade F, representing 3.23% (3/93). Based on the content of the white paper on the GTT method, it was calculated that the number of ADE occurrences in 1,000 patient-days was 22.90 ((93/4,062)*1,000)); the number of ADE occurrences in 100 patients was 19.38, and the incidence rate of ADEs in patients was 13.54%.

### 3.5 Classes of drugs involved in ADEs

**FIGURE 2:**
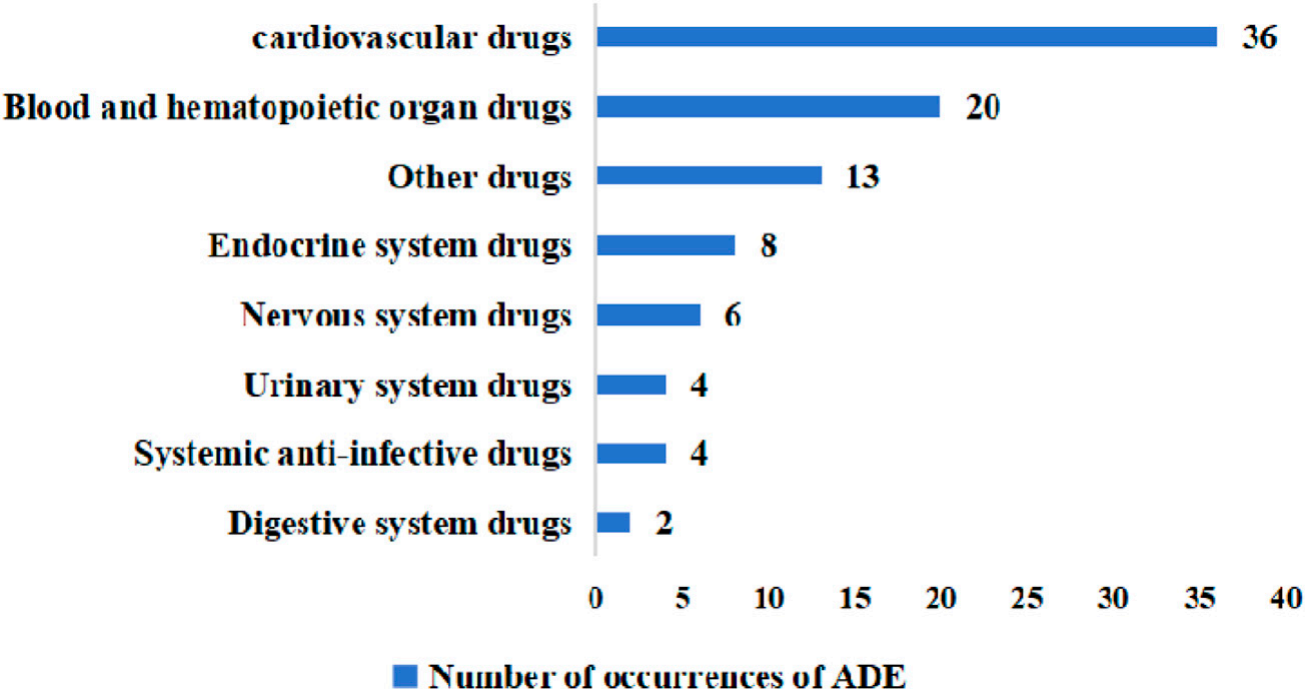
Instances of ADEs in relevant drug classes. ADE, adverse drug event.

Categorizing the drugs involved, the highest number of ADE cases occurred in the cardiovascular drugs, involving 16 drugs such as spironolactone, furosemide, digoxin, torasemide, etc., with a total of 36 cases, accounting for 38.71% (36/93). The next type was blood and hematopoietic organs drugs with 20 cases, accounting for 21.51% (20/93), and the main drugs involved were clopidogrel sulfate tablets, warfarin, and heparin. In third place was other types, involving drugs such as potassium chloride and vitamin K1 injection in 14 cases or 13.98% (13/93). There were also drugs involving the endocrine system in 8 cases, the nervous system in 6 cases, systemic anti-infectives and the urinary system in 4 cases each, and the digestive system in 2 cases (Figure 2).

### 3.6 Organ-system involvement in ADE

**FIGURE 3:**
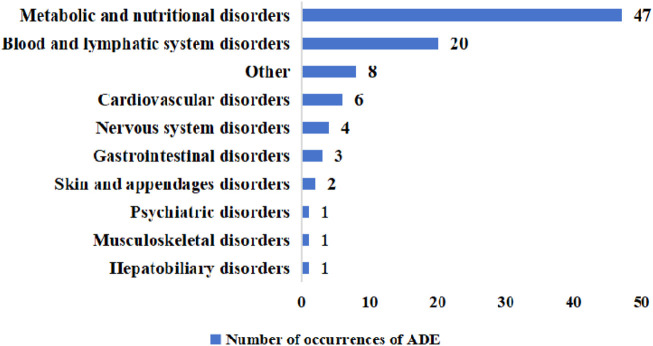
Organs-systems involved in the occurrence of ADE. ADE, adverse drug event.

In this study, the highest occurrence of ADE involving organ-systems was metabolic and nutritional disorders with 47 cases accounting for 50.54% (47/93), which included hypokalemia, hyperkalemia, hyponatremia, hypernatremia, hypoglycemia, and renal dysfunction. This was followed by hematologic and lymphatic system including leukopenia, thrombocytopenia and coagulation disorders that occurred in 20 cases that accounted for 21.51% (20/93). Other types of edema occurred third with 8 cases representing 8.60% (8/93). Cardiovascular system occurred in 6 cases, gastrointestinal damage in 4 cases, neurological damage in 3 cases, skin and its accessories in 2 cases, and hepatobiliary, psychiatric, and musculoskeletal damage in 1 case each (Figure 3).

### 3.7 Factors affecting the occurrence of ADEs detection

The basic information of the patients was used to determine whether there were factors affecting the detection of ADE. The results showed that age, number of days of hospitalization, number of medications and number of positive trigger detections were all associated with the occurrence of ADE (*P* < 0.05) (Table 4).

**TABLE 4:** Factors influencing basic patient information for detecting ADEs.

Variable	Patients with ADE (n = 65)	Patients without ADE (n = 415)	Total	*P* value	χ^2^/Z value
Gender					
Male	32 (12.17%)	231 (87.83%)	263	0.333	0.939
Female	33 (15.21%)	184 (84.79%)	217		
Age (years)					
65–75	17 (5.61%)	286 (94.39%)	303	0.000	−6.809
76–85	28 (19.05%)	119 (80.95%)	147		
≥86	20 (66.67%)	10 (33.33%)	30		
Hospitalization days					
2–13	51 (11.92%)	377 (88.08%)	428	0.033	−2.129
13–24	14 (26.92%)	38 (73.08%)	52		
Number of medications					
5–15	29 (11.11%)	232 (88.89%)	261	0.037	−2.082
16–26	27 (15.25%)	150 (84.75%)	177		
≥27	9 (21.95%)	32 (78.05%)	41		
Trigger positives detection					
0	14 (3.92%)	343 (96.08%)	357	0.000	110.125
≥1	51 (41.46%)	72 (58.54%)	123		
Number of chronic diseases					
2–3	56 (12.17%)	404 (87.83%)	460	0.522	−0.641
≥4	9 (45.00%)	11 (55.00%)	20		

Binary logistic regression was used to further analyze four factors associated with the occurrence of ADE: age, number of days of hospitalization, number of medications and number of triggers detected positive. The results showed that age and number of triggers detected positive were risk factors for the occurrence of ADE in elderly hospitalized patients with multiple chronic diseases (*P* < 0.05) (Table 5).

**TABLE 5:** Results of binary logistic regression.

Risk factor	*P* value	OR	95%CI
Age	0.000	3.565	2.181–5.826
Days of hospitalization	0.601	1.289	0.498–3.331
Number of medications	0.355	1.266	0.768–2.085
Trigger positives detection	0.000	14.317	7.277–28.168

OR, The Odds Ratio.

The larger the Odds Ratio (OR) value, the higher the likelihood of the risk factor. As shown in Table 5, the largest OR value was the number of positive trigger detections, followed by age, and the two variables are risk factors.

**FIGURE 4:**
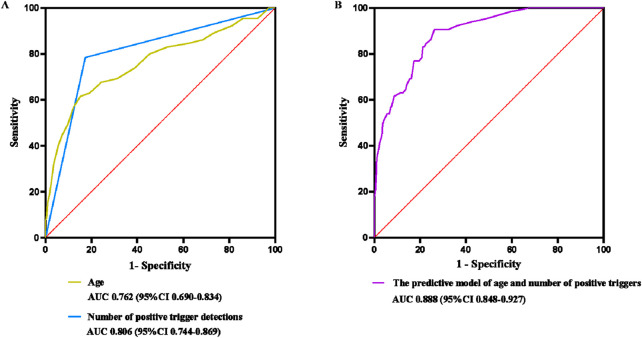
ROC plot of the risk of ADE occurrence. ROC: Receiver Operating Characteristic; ADE: adverse drug event. **(A)** ROC curve of risk factors on the occurrence of ADE. **(B)** ROC plot of the predictive model of the risk of ADE occurrence.

The two risk factors: age and the number of positive trigger detections, were used to predict the optimal equation using the ROC curve. The results of the predictive model showed that AUC = 0.888 (95%CI: 0.848–0.927). AUC >0.75 indicates that the model shows good discrimination (Figure 4).

## 4 Discussion

The PPV was 41.46% in our study. Although there is no universally accepted definition to distinguish between’good’ and ‘poor’ performance of a trigger tool—depending on its intended use——a PPV ≥20% is generally considered good (Landis et al., 1977). Our PPV was relatively high compared to other studies on the elderly 32.45%–41.8% (Zhang and Wang, 2021; Noorda et al., 2022), confirming the effectiveness and accuracy of the triggers established in this study.

### 4.1 Trigger detection analysis

As shown in Table 2, the items “TSH< 0.56 mU/L,TH > 152.52 nmol/L” and “TSH >5.91 mU/L,TH < 69.97 nmol/L”, the two triggers that test positive but no ADE occurred. This may be attributed to other factors, such as pituitary dysfunction and autoimmune diseases. The reason why ADE also did not occur for “use of glycerol enema/lactulose oral solution” may be (1) age-related: the older the age, the slower the gastrointestinal peristalsis function; (2) pre-existing disease of the gastrointestinal tract itself. And “hemoglobin >175 g/L” may be caused by environmental factors, lung disease, kidney disease. “Transferred to the intensive care unit/re-admitted within 30 days” may be due to the seriousness of the disease or the difference between the previous diseases that led to re-hospitalization. This reflects a common challenge in the implementation of GTT: there is no uniform standard for laboratory trigger thresholds. Too low a threshold can lead to frequent false positives and alert fatigue, while too high a threshold may result in the omission of ADE (Li et al., 2023). According to the relevant literature, the occurrence of the above situations may affect the detection of the triggers, so these five triggers need to be further optimized and improved (Huang and Wen, 2021).

Valkonen et al. (2023) investigated the evaluation of GTT as a drug safety tool for detecting ADEs and showed that the number of triggers in the GTT drug module was associated with the risk of ADEs and that modification of the GTT was able to prevent ADEs and provide more reliable data. Therefore, the triggers that were not detected as positive in this study require further modification, including “use of flumazenil”, “use of naloxone”, “abrupt discontinuation of medication”. Five triggers, including “use of flumazenil”, “use of naloxone”, “abrupt discontinuation of medication”, “dehydration”, and “urinary retention”, could be selected for deletion due to their low utilization and incidence. The remaining 6 triggers, including “positive fecal *C. difficile* test”, “excessive sedation/hypotension/falls”, “use of protamine sulfate”, “use of epinephrine or glucocorticoids”, “vancomycin blood drug concentration >10 mg/L″ and “gentamicin blood drug concentration >10 mg/L″ had a lower utilization rate and incidence rate, but should be retained because of their serious outcomes when ADEs occur.

Although this study focuses on the high-risk elderly patients with multiple chronic diseases, the core laboratory and symptom triggers (e.g., blood glucose <3.9 mmol/L) of the GTT remain valuable for monitoring in patients without multiple medications or with a single disease. Despite the low complexity of medication regimens in such patients, age-related decline in liver and kidney function and changes in drug metabolism can still lead to ADEs (Zhang and Wang, 2021). Optimisation of GTT has the potential to be extended to a wider elderly population.

### 4.2 ADE determination and grading situation analysis

When applying the Naranjo scale, item 5 was challenging to completely exclude other causes (e.g., co-morbidities or age-related changes) in the case of polypharmacy; also, item 6 was uniformly rated as “0” due to ethical constraints. This is consistent with the limitations of the Naranjo scale noted by Kaur et al. (2024), but more complete clinical data (e.g., continuous vital signs, multidisciplinary records) in inpatients partially compensated for missing data in outpatient studies.

The Naranjo method may result in a high incidence of reported ADEs and does not allow for the evaluation of two or more drug interactions. Therefore, the Naranjo scale suggests a score of 5-8 on the “probable” scale (Naranjo et al., 1981). In this study, the ADE was mainly focused on the score of “possible” 1-4, and only 65 out of 480 cases were determined to have an ADE, which is a small number with a low incidence rate of only 13.54% of the total sample. This may be due to the fact that some of the medications in the sample cases were present in the medical prescription, but there was no indication in the case as to why they were used or the reason for their own disease affecting the detection of the trigger, which ultimately led to the identification of fewer cases of ADE occurrence. According to Parrinello et al. (2019), who evaluated the development and implementation of the GTT method in a large health system in Sicily, the overall incidence of in-hospital ADEs was 9.2%, and the incidence of ADEs in 44 hospitals ranged from 7% to 51%, which makes the GTT method effective in monitoring ADEs. Accordingly, the incidence of ADEs in this paper is within a reasonable range.

The study with NCC MERP medication error grading criteria had no G-I grades and mostly focused on grade E. It may be due to the low use of high-risk medications or to the fact that no serious ADEs occurred in the sample cases included. However, since the NCC MERP medication error grading standard relies on subjective grading, which may lead to the omission of high-level events, and the standard itself cannot analyse the systemic causes, future research needs to combine multidimensional tools to address the design of preventive mechanisms.

### 4.3 Relevant drug classes and organ-system analyses involved in ADEs

Compared with the 2023 national adverse drug reaction report, the proportion of cardiovascular system drugs was much higher than the 7.9% in the report; the proportion of systemic anti-infective drugs was much lower than the 30.5% in the national adverse drug reaction report (National Center for ADR Monitoring, 2024). The incidence of ADE caused by cardiovascular system drugs was as high as 38.71%, which may be due to the fact that the subjects of this study were elderly inpatients with multiple chronic diseases, and the incidence of cardiovascular disease was the highest (Chen et al., 2018). Several studies (Chan et al., 2001; Zhang and Wang, 2021) have also shown that cardiovascular drugs are the most common causative drugs of ADE in the elderly population. Cardiovascular diseases often involve multiple organs and have complex pathomechanisms that require the use of a wide variety of drugs, resulting in a high incidence of ADEs (Amrouch et al., 2025; Marović et al., 2024). While systemic anti-infectives also account for a high percentage of elderly patients, but in this study the incidence of ADEs was low, probably because fewer selected cases had ADE caused by systemic anti-infective drugs (Chen et al., 2025). In addition, the highest number of cases of ADE involving organ-systems in this study was metabolic and nutritional disorders. Hypokalemia occurred in the highest number of ADE cases, accounting for 25.81% of the total ADE cases. This is consistent with the literature that hypokalemia is a common type of ADE (Härkänen et al., 2015).

### 4.4 Analysis of risk factors affecting ADE

Analysis of risk factors by binary logistic regression revealed that there was no significant difference between the number of days of hospitalization, number of medications and the number of chronic diseases (*P* > 0.05). It is possible that the variable was randomly sampled with a low number of cases at a certain stage (Zhang et al., 2023). In addition, according to the relevant literature (Rasool et al., 2023; Saedder et al., 2015), the most common risk factor for elderly patients is the number of medication scales. Whereas in this study the number of medications was not a risk factor, probably due to the influence of other variables, which resulted in insignificant differences in the number of medication scales used. In this study, age was the risk factor, and Zhang et al. studied the factors affecting the risk of combined cardiovascular disease in elderly patients with chronic diseases and also found that age was the most important and uncontrollable factor in this group of patients (Zhang et al., 2025), which is consistent with this study.

### 4.5 Strengths and limitations

Strengths: This study focuses on the use of the GTT method to effectively detect ADEs in high-risk elderly inpatients with multiple chronic diseases. The advantages of GTT include low labour intensity and strong resource compatibility. Only two reviewers are needed to screen all cases in the hospital, whereas prospective tools such as MISO require a dedicated team to respond 24 h a day (Chakrabarti and Kaur, 2024). In the future, GTT screening and ISO intervention may be considered.

Limitations: In this study, the ADR causality assessment in this study relied on the Naranjo scale, which has limited ability to detect drug interactions; also, retrospective case records may be incomplete. Although this deficiency is remedied by pharmacovigilance records and multidisciplinary visit records, the future development of geriatric-specific assessment tools remains an urgent need. Secondly, some triggers need to be further modified for the GTT method of monitoring ADE in elderly inpatients with multiple chronic diseases, a dynamic threshold strategy should be considered for laboratory triggers in the future; the drug class (systemic anti-infective drugs) and the influencing factors (number of days in hospital, number of chronic diseases, and number of medications used) need to be further investigated and analysed by enlarging the sample size in order to make a better judgement of the effect.

## 5 Conclusions and application prospects

### 5.1 Conclusions

In summary, the GTT method was used in this study to monitor ADEs in the high-risk elderly inpatients with multiple chronic diseases, and its application was evaluated and analysed. The results showed that the GTT method had relatively high PPV, sensitivity, and specificity, and was feasible and reliable, indicating the effectiveness and practicality of the trigger, which can effectively detect ADEs in this type of patient.

### 5.2 Application prospects

Clinical pharmacists can use the results of this type of research as a starting point for their work. For entries with high PPV triggers, when developing medication regimens, they should fully consider the management and preventive measures for ADEs that may occur, thereby ensuring patient medication safety. In future work, we will consider developing a dynamic trigger system based on machine learning, which can analyse electronic medical record medication records, laboratory data, etc., In real time to achieve ADE risk grading warnings. Additionally, we can convert the high-risk patterns identified by GTT into specific intervention measures within the MISO (monitoring, instructions, the start-low/go-slow approach, and omission) framework (Chakrabarti and Kaur, 2024). For example, in the ‘Omission’ phase, high-risk drug combinations can be prioritised for exclusion, achieving a closed-loop management system from risk warning to precise medication administration. These improvements are expected to upgrade the current ADE monitoring model from passive identification to proactive prevention and control, providing more comprehensive safeguards for medication safety in elderly patients with multiple chronic conditions.

## Data Availability

The original contributions presented in the study are included in the article/Supplementary Material, further inquiries can be directed to the corresponding author.
