# Supplementary material for: Application and evaluation of the global trigger tool approach to adverse drug event monitoring in the high-risk elderly inpatients with multiple chronic diseases
Source: Front Pharmacol. 2025 Jun 20;16:1594176. doi: 10.3389/fphar.2025.1594176 (PMC12226562; doi:10.3389/fphar.2025.1594176)
Supplement: Supplementary file 1 [file DataSheet1.PDF]

TABLE S1. Naranjo Rating Method

No.	Rating item	Yes	No	Unknown
1	Has the adverse reaction been reported previously	+1	0	0
2	Did the adverse reaction occur after use of the suspected drug	+2	-1	0
3	Whether adverse effects improved with drug discontinuation or antagonist use	+1	0	0
4	Whether the adverse effects recurred when the suspected drug was taken again	+2	-1	0
5	Is there any other reason for the adverse reaction	-1	+2	0
6	Whether the adverse reaction can recur when placebo is given	-1	+1	0
7	Whether the blood concentration is a toxic concentration	+1	0	0
8	Whether the seriousness of adverse effects is related to dose increase or decrease	+1	0	0
9	Whether the patient has had similar adverse effects from previous use of the same or similar medications	+1	0	0
10	Whether the adverse reaction was confirmed by objective examination	+1	0	0

TABLE S2. Characteristics of 95 Adverse Drug Events (ADEs) Stratified by Naranjo Causality Assessment

Positive trigger	Adjudication of ADE	Classification of ADEs
restlessness	possible	E
Joint pain	possible	E
Rash/Itch	certain	E
Rash/Itch	certain	E
Digoxin Blood Concentration > 2 ng/ml	likely	E
Digoxin Blood Concentration > 2 ng/ml	likely	E
Use of metoclopramide	possible	E
Use of metoclopramide	possible	E
Headache	possible	E
Dizziness	possible	E
edema	possible	E
edema	possible	E
edema	possible	E
edema	possible	E
edema	possible	E
edema	possible	E
edema	possible	E
edema	possible	E
Cognitive impairment	doubtful	E
montelukast/bifidobacterium trifidum/bacillus subtilis/oral metronidazole	possible	E
montelukast/bifidobacterium trifidum/bacillus subtilis/oral metronidazole	doubtful	E
PLT＜125×10^9/L	possible	E
PLT＜125×10^9/L	possible	E
PLT＜125×10^9/L	possible	E
PLT＜125×10^9/L	possible	E

PLT＜125×10^9/L	possible	E
PLT＜125×10^9/L	possible	E
TC＞5.2 mmol/L	possible	E
TC＞5.2 mmol/L	possible	E
TC＞5.2 mmol/L	possible	E
TC＞5.2 mmol/L	possible	E
TC＞5.2 mmol/L	possible	E
TC＞5.2 mmol/L	possible	E
WBC＜3.5×10^9/L	possible	E
WBC＜3.5×10^9/L	possible	E
WBC＜3.5×10^9/L	possible	E
Na＜137 mmol/L	possible	E
Na＜137 mmol/L	possible	E
Na＜137 mmol/L	possible	E
Na＜137 mmol/L	possible	E
Na＞147 mmol/L	possible	E
Na＞147 mmol/L	possible	E
Na＞147 mmol/L	possible	E
Na＞147 mmol/L	possible	E
K＜3.5 mmol/L	possible	E
K＜3.5 mmol/L	possible	E
K＜3.5 mmol/L	possible	E
K＜3.5 mmol/L	possible	E
K＜3.5 mmol/L	possible	E
K＜3.5 mmol/L	likely	F
K＜3.5 mmol/L	likely	E
K＜3.5 mmol/L	likely	E
K＜3.5 mmol/L	possible	E
K＜3.5 mmol/L	possible	E
K＜3.5 mmol/L	possible	E
K＜3.5 mmol/L	possible	F

K＜3.5 mmol/L	possible	E
K＜3.5 mmol/L	possible	E
K＜3.5 mmol/L	possible	E
K＜3.5 mmol/L	possible	E
K＜3.5 mmol/L	possible	E
K＜3.5 mmol/L	possible	E
K＜3.5 mmol/L	possible	E
K＜3.5 mmol/L	possible	E
K＜3.5 mmol/L	possible	E
K＜3.5 mmol/L	possible	E
K＜3.5 mmol/L	possible	E
K＜3.5 mmol/L	possible	E
K＞5.3 mmol/L	possible	E
K＞5.3 mmol/L	possible	E
K＞5.3 mmol/L	possible	E
ALT＞35 U/L；AST＞40 U/L；ALP＞125 U/L；TBIL＞23 μmol/L	possible	F
Use of vitamin K	possible	E
CK＞164 U/L；BUN＞9.5 mmol/L；SCr(male)＞111 μmol/L；SCr(female)＞81 μmol/L	possible	E
CK＞164 U/L；BUN＞9.5 mmol/L；SCr(male)＞111 μmol/L；SCr(female)＞81 μmol/L	possible	E
CK＞164 U/L；BUN＞9.5 mmol/L；SCr(male)＞111 μmol/L；SCr(female)＞81 μmol/L	possible	E
CK＞164 U/L；BUN＞9.5 mmol/L；SCr(male)＞111 μmol/L；SCr(female)＞81 μmol/L	possible	E
CK＞164 U/L；BUN＞9.5 mmol/L；SCr(male)＞111 μmol/L；SCr(female)＞81 μmol/L	possible	E
BG＜3.9 mmol/L	possible	E
BG＜3.9 mmol/L	possible	E
BG＜3.9 mmol/L	possible	E

BG＜3.9 mmol/L	possible	E
BG＜3.9 mmol/L	possible	E
BG＜3.9 mmol/L	possible	E
BG＜3.9 mmol/L	possible	E
INR＞1.2 seconds	possible	E
INR＞1.2 seconds	possible	E
APTT＞39 seconds	possible	E
APTT＞39 seconds	possible	E
APTT＞39 seconds	possible	E
APTT＞39 seconds	possible	E
APTT＞39 seconds	possible	E
APTT＞39 seconds	possible	E
APTT＞39 seconds	possible	E
PLT＜125×10^9/L	possible	E

Two cases classified as ‘doubtful’ were excluded based on the Naranjo criteria.
